# 

**Published:** 1850-07-01

**Authors:** 


### CONTENTS.

PAGE

Religious Insanity . . . . . . . . . . . . . . . . 283

Causes, Cure, and Prevention of Idiocy . . . . . . . . . 292

Dr. Davey's Mental Pathology . . . . . . . . . . . . 322

The Pennsylvania Hospital for the Insane . . . . . . . . 339

Lectures on the Pathological Appearances observed in the Bodies of the Insane. No. II. By Dr. John Hitchman 362

The Physiology of the Human Brain . . . . . . . . . 373

Colney Hatch Asylum . . . . . . . . . . . . . . 384

Confessions of a Patient after Recovering from an Attack of Lunacy . . . . . . . . . . . . . . . . 388

The French Law of Lunacy . . . . . . . . . . . . 400

Mr. Dyce Sombre on the English Law of Lunacy . . . . 409

Case of Pyromania . . . . . . . . . . . . . . . 419

Miscellaneous Notices . . . . . . . . . . . . . . 420

Appointment . . . . . . . . . . . . . . . . . 424

To Correspondents . . . . . . . . . . . . . . . 424

